# Glucose-6-phosphate dehydrogenase deficiency induced hemolytic anemia and methemoglobinemia: a case report in a 7 -year-old female patient

**DOI:** 10.1186/s13052-025-02051-2

**Published:** 2025-08-21

**Authors:** Adalgisa Fastuca, Antonio Vergori, Giuseppe Robustelli, Chiara Piccolo, Maria Ragazzo, Maddalena Marinoni, Massimo Agosti

**Affiliations:** 1 University of Insubria, Varese, Italy; 2 Pediatric Unit, Maternal and Child Department, “F. Del Ponte” Hospital, Varese, Italy; 3 Pediatric Oncoematology Unit, “F. Del Ponte” Hospital, Varese, Italy

**Keywords:** Case report, Hemolytic anemia, Glucose-6-phosphate dehydrogenase (G6PDH) deficiency, Methemoglobinemia, Child

## Abstract

**Background:**

Patients affected by glucose-6-phosphate dehydrogenase (G6PDH) deficiency are often asymptomatic until an oxidative stress occurs, causing acute hemolytic anemia. The coexistence of hemolytic crisis secondary to G6PDH deficiency and methemoglobinemia is an already known phenomenon, especially after the ingestion of fava beans. While past literature described this association primarily in adult patients, it remains an unusual finding in pediatric population. Our patient's age, as long as her gender, and a negative family history represent, indeed, the peculiarity of what we described.

**Case presentation:**

We present the case of a 7-year-old female patient with a clinical history of hyperpyrexia, several episodes of yellowish vomit, hypercromic urine, loose stools, asthenia with jaundice. At home paracetamol and a single dose of ibuprofen were administered. No recent history of fava bean ingestion or relevant events were reported in physiological and pathological anamnesis. Family history was negative for hematological comorbidities. Blood tests performed at the emergency room showed a picture of severe anemia with negative direct and indirect Coombs tests, mild acute renal failure, increased inflammation markers and a methemoglobin level equal to 13.7% on blood gas analysis. The detection of vital parameters showed O2-Sat equal to 75% without signs of respiratory distress. A broad-spectrum antibiotic therapy with ceftriaxone and oxygen-therapy were administered, hospitalization was then arranged. Immune-mediated anemia and lymphoproliferative diseases were excluded. G6PDH dosage was requested, resulting indicative of deficiency. Fecal virus testing revealed a positivity for Norovirus. Transfusions of red cell concentrates (RCC) were performed, and the methemoglobin value gradually decreased with stabilization of the hemoglobin, so that methylene blue therapy was not administrated. The patient’s clinical conditions improved in almost 5 days.

**Conclusions:**

When G6PDH deficiency and methemoglobinemia coexist, a prompt diagnosis is essential. The administration of methylene blue, therapy of choice for the treatment of methemoglobinemia, in fact, may cause a worsening of hemolytic crises in patients affected by G6PDH deficiency. Considering our patient’s clinical features, not clearly evocative of G6PDH deficiency, this case represented a challenge for both diagnosis and treatment, reminding to always consider G6PDH deficiency in case of hemolytic anemia associated with methemoglobinemia.

## Background

G6PDH deficiency represents the most common erythrocyte enzyme defect, counting almost 400 million people affected worldwide. Its prevalence is consistent especially in African and Asian countries, and in the Mediterranean area [1, 2].

The specific gene encoding for G6PDH is located on the long arm of the X chromosome (Xq28) and carries out more than 400 enzyme variants with different functional and biochemical features.

In detail, GdA- (African) and GdB- (Mediterranean) represent the most frequent variants. Particularly, GdB- variant, because of its shorter half-life, which makes the deficiency detectable even in younger erythrocytes, is related to a worse prognosis [2].

Classification and main clinical manifestations of G6PDH deficiency are detailed in Table 1, [2, 3] and Table 2, [2].

**Table 1:** WHO Classification of G6PDH deficiency

WHO Class	Enzyme Activity Level	Clinical Severity
*Class I*	1- 5%	Severe deficiency
*Class II*	5—10%	Severe to Moderate deficiency
*Class III*	10—60%	Moderate deficiency
*Class IV*	60—100%	Mild to no deficiency
*Class V*	> 100%	Increased enzyme activity

**Table 2:** Main clinical manifestations of G6PDH deficiency

Syndrome	
Favism	Classically triggered by the ingestion of fava beans, may also occur following drug administration or during intercurrent infections (e.g., salmonellosis, pneumoniae, sepsis or viral hepatitis)
Neonatal jaundice	Early- onset jaundice in newborns
Chronic non-spherocytic hemolytic anemia	Persistent hemolytic anemia not associated with spherocytosis

Patients affected by G6PDH deficiency are often asymptomatic until an oxidative trigger occurs, causing clinical manifestations due to acute hemolytic anemia. G6PDH has indeed a primary role in the first biochemical reaction of pentose phosphate pathway (PPP), conditioning the synthesis of reducing equivalents, strictly related to Nicotinamide adenine dinucleotide phosphate (NADPH) production [2]. In detail, this molecule seems to be essential for glutathione (GSH) metabolism which plays an important role in antioxidant defense, nutrient metabolism, and cellular activity regulation. A deficiency in GSH contributes to hemoglobin denaturation and erythrocyte membrane proteins oxidation, causing hemolysis.

Although G6PDH is present within all cells, its deficiency gets evident only for erythrocytes because they cannot activate other pathways to produce NADPH as they do not possess any nucleus.

Favism represents the most common clinical manifestation. It usually occurs as acute hemolytic anemia with a variable timing of clinical presentation (from hours to several days after trigger exposition). It is usually characterized by the onset of worsening pallor, jaundice and hypercromic urine. In addition, it is possible to notice nonspecific signs and symptoms such as abdominal pain, hepatosplenomegaly**,** polypnea, tachycardia, hypotension and, especially in case of hemoglobinuria, acute renal failure should be considered.

Blood tests typically reveal a picture of normocytic anemia with reticulocytosis and leukocytosis with a reactive neutrophilia, unconjugated hyperbilirubinemia and often a moderate increase in transaminases level [2].

Direct and indirect Coombs tests are negative. Microscopic analysis of the peripheral blood smear may show abnormal erythrocyte fragments secondary to hemolysis.

During the crisis, reticulocytosis may hide the enzyme deficiency, because young erythrocytes usually have a normal concentration of G6PDH. However, in subjects with homo- or hemizygous mutations, the enzymatic level can show the deficiency even during the acute phase of the disease, making the diagnosis possible [2].

Differential diagnosis, not always easy to perform, place a crucial role in the diagnostic process; autoimmune hemolytic anemia, other hereditary enzyme deficiencies such as pyruvate kinase deficiency, spherocytosis and in some cases the hemolytic-uremic typical and atypical syndromes should be considered.

In patients affected by G6PDH deficiency, acute hemolysis may coexist with the finding of methemoglobinemia. Generally, methemoglobinemia, an anomalous oxidized type of hemoglobin in which the ion linked to the heme group switches from the ferrous (2 +) to the ferric (3 +) oxidation state, making the protein unable to transport oxygen to tissues, forms as an acquired process secondary to exposure to oxidizing factors such as drugs or chemical substances, although it may rarely be related to a congenital etiopathogenesis.

The association between G6PDH deficiency and methemoglobinemia depend on the fact that both phenomena, aside from sharing the same triggering agents, sustain each other: the poor availability of NADPH typical of patients with a G6PDH deficiency modifies the main pathway involved in the reduction of methemoglobin [4, 5]. The need for treatment of methemoglobinemia depends on symptoms and blood levels of methemoglobin. The first step is always represented by the elimination of the triggering agents. In case of symptoms persistence or in asymptomatic patients with methemoglobin levels > 30%, it is necessary to administrate methylene blue at 1–2 mg/kg. However, it is essential to identify cases in which methemoglobinemia coexists with G6PD deficiency as the intake of methylene blue may favor hemolysis, worsening the clinical manifestations [6].

While past literature described this association primarily in adult patients [5, 7–17], it was highlighted in a limited number of pediatric studies [6, 17–24]**.**

## Case presentation

V. is a 7-year-old female patient, admitted to our pediatric clinic with a clinical history characterized by hyperpyrexia for approximately 2 days, preceded by several episodes of vomiting, with subsequent emission of hypercromic urine, two episodes of loose stools without visible blood, and profound asthenia associated with a pale-icteric skin colour. Only paracetamol and a single dose of ibuprofen, never taken before, were administered at home. Family history for hematological pathologies was negative. Non-consanguinity of parents was reported. Physiological anamnesis did not report any perinatal issues.

Our patient, as her parents, had never eaten fava beans or other legumes in their lifetime.

Upon arrival at the Pediatric Emergency Room the patient appeared asthenic, pale, still responsive to simple questions. The cardio-thoracic and abdominal objectivity was within normal limits. The vital parameters detection showed an Oxygen Saturation (O_2_-Sat) of 75% in room air, poorly responsive to the administration of oxygen therapy, a respiratory rate of 20 breaths/min and a heart rate of 120 bpm.

The blood tests performed showed a severe anemia (Hb 6.3 gr/dL, n.v. 11.5–14.5 gr/dL), an increase in inflammatory markers (WBC 21,840/mmc, n.v. 5,000–12,000/mmc; CRP 50.6 mg/L, n.v. < 8 mg/L), a normal platelet count (PLT 190,000/mmc, n.v. 150,000–450,000/mmc), high ferritin levels (15,648 ng/mL, n.v. 6–320 ng/mL), an increased total bilirubin level (3.2 mg/dL, n.v. < 1.20 mg/dL with indirect bilirubin level of 2.9 mg/dL, n.v. < 0.85 mg/dL), an increased creatinine level (0.65 mg/dL, n.v. 0.34–0.53 mg/dL), LDH dosage was beyond the upper limit of detection of the laboratory test (first value available equal to 2,531 U/L, n.v. < 308 U/L). Reticulocyte absolute count was equal to 69,000/mmc (n.v. 22,000–92,000/mmc).

Direct and indirect Coombs tests resulted negative. Myoglobin and haptoglobin levels were normal. On the peripheral blood smear erythroblasts, myelocytes and meta myelocytes were detected; no schistocytes were present. The capillary blood gas test and subsequently arterial blood gas test showed a level of methemoglobinemia of 13.7% (n.v. < 1%) with a pCO2 of 32 mmHg (n.v. 35–45 mmHg) and a pO2 of 90 mmHg (n.v. 75–100 mmHg).

Standard urine analysis revealed a pH 6.5 (n.v. 4.5–8), specific gravity (SG) equal to 1015 (n.v. 1005–1030), high levels of hemoglobin with 150 red blood cells/μL (n.v. < 20 red blood cells/μL) and increased proteins (147 mg/dL, n.v. < 30 mg/dL).

An ECG and a cardiological evaluation were performed, showing normal results.

Infusion with saline solution and antibiotic therapy with ceftriaxone iv were administrated, considering the mild increase in inflammatory markers and the patient's overall clinical condition.

Hospitalization to our pediatric ward was then arranged and, after pre-transfusion serologies, as the autoimmune origin of the hemolytic anemia could not be excluded with certainty (the routine direct and indirect Coombs tests may not be accurate in cases of cold agglutinin disease), two transfusions of RCC were performed with a dose of 5 ml/kg. Steroid therapy with methylprednisolone was started until cold hemagglutinins research resulted negative. In addition, as blood tests showed a progressive decline in platelets count and a persistent hyperferritinemia, before steroid administration, immunophenotype on peripheral blood, bone marrow fine needle aspiration and bone marrow biopsy were performed, confirming the absence of blasts and showing no signs of hematophagocytosis.

Because of the persistence of symptoms, despite the acute hemolysis condition and the risk of a false negative result, quantitative G6PDH dosage was performed and resulted indicative of deficiency (equal to 7.1 U/g of hemoglobin with physiological range 10.1–14.2 U/g of hemoglobin).

**Fig. 1:**
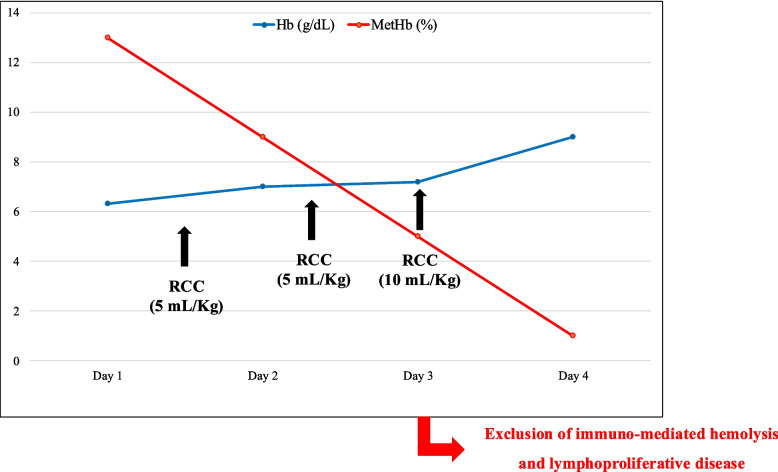
Hb and MetHb variations according to RCC trasfusions

On the third day of hospitalization, considering this result, an RCC transfusion at the dosage of 10 ml/kg was administrated and well tolerated. (Hb pre-transfusion 7.0 gr/dL) (Fig. 1).

To complete the diagnostic picture, further investigations were performed:

- Abdominal ultrasound excluded the presence of organomegaly or abdominal masses and reported abundant gallbladder bile sediment.
- Chest X-Ray resulted negative for the presence of mediastinal masses.
- Serologies for EBV, CMV, Parvovirus B19 and Mycoplasma pneumoniae were negative for acute infection
- Fecal viruses research resulted positive for Norovirus
- All blood-, stool-, throat- and urine cultures resulted negative.

The subsequent blood tests showed a progressive improvement in the hemoglobin value with adequate reticulocytosis (reticulocyte absolute value: 178,000/mmc), an increase in platelets and a normalization of white blood cells count.

Blood gas analysis showed a progressive reduction of methemoglobin levels; therefore, oxygen therapy was suspended. A progressive normalization of bilirubinemia, a reduction in ferritin and LDH levels were also observed. Renal function markers showed an initial increase in creatinine level (maximum value 0.9 mg/dl on the second day of hospitalization), associated with a contraction of diuresis (minimum value equal to 0.7 ml/kg/h, n.v.1–2 ml/Kg/h), with a gradual normalization of creatinine and the appearance of polyuria post-acute renal failure (maximum value equal to 3 ml/kg/h). No diuretic therapy was required. Arterial blood pressure always remained within normal limits. Urinary tests revealed the presence of tubular proteinuria with maximum uPr/uCr equal to 3.9 mg/mg (n.v. < 0.2 mg/mg) and maximum urinary beta-2-microglobulin/uCr equal to 30 mcg/mg (n.v. < 4 mgc/mg).

Signs of tubular distress also appeared: mild hypokalemia (minimum value 3.1 mEq/L, n.v. 3.5–5.2 mEq/L), hypouricemia (1.8 mg/dL, n.v. 2.4–5.7 mg/dL), an increase in the fractional excretion of sodium (maximum FENa 2.8%, n.v. < 1%) and transient glycosuria not associated with hyperglycemia.

These data were compatible with acute tubular necrosis probably secondary to Norovirus infection. In the following days a normalization of the hydro-electrolytic state was reached.

During the hospitalization we assisted to a progressive improvement of asthenia, a resolution of hyperpyrexia with stable apyrexia from the third day of hospitalization associated with resumption of nutrition and a normalization of alvus disorders.

The G6PDH dosage in both parents was indicative of deficiency (mother 0.3 U/g of hemoglobin, father 0.7 U/g of hemoglobin, physiological range 10.1–14.2 U/g of hemoglobin).

Considering the results of the patient's quantitative enzymatic assay, which revealed a marked G6PDH deficiency, no further genetic analysis was performed, as this is not part of the routine diagnostic workflow at our centre.

## Discussion and Conclusions

In our case report two main agents were found to be the possible trigger of the acute hemolytic crisis: on the one hand the intercurrent Norovirus infection, detected through fecal sample analysis, on the other hand the administration of ibuprofen, which belongs to the category of drugs with an intermediate risk of causing hemolysis in patients suffering from G6PDH deficiency.

Furthermore, an unusual fact is represented by sex and age of onset, without a significant remote physiological and pathological anamnesis. In fact, taking into account the hereditary etiopathogenesis of the disease, heterozygous females usually present intermediate levels of erythrocytic G6PDH between healthy subjects and deficient homozygote males. This phenomenon is explained, similarly to other genetic diseases with an X-linked hereditariness, because of the functional inactivation of one of the two X chromosomes in females. Since inactivation is a random process, although heterozygous females generally have the half quantity of the normal level of G6PDH, it may happen, as in our case, that preferential inactivation of the healthy X chromosome occurs.

In this context, comprehensive genetic testing not only of the patient but also of both parents may help to better define specific genotype–phenotype correlations. Evidence from the literature on other X-linked disorders, in fact, has shown that, even within genetically and clinically well-characterized syndromes, the presence of atypical or unexpected clinical features may sometimes be attributed to additional genetic variants, often identified in one of the parents [25, 26].

In addition, it is not uncommon in patients with G6PDH deficiency to have a history of severe neonatal pathological jaundice with the need of phototherapy or exchange transfusion, absent in our patient’s perinatal anamnesis.

An unusual data was also represented by the family clinical history, completely negative for hematological and genetic diseases, despite the very low G6PDH levels in both parents.

Blood gas tests performed in our case highlighted a high percentage of methemoglobin (MetHb) at onset. Patients with abnormal levels of methemoglobin at blood gas tests usually have a reduced oxygen saturation on pulse oximeter and a falsely high oxygen saturation at arterial blood gas analysis. The need for treatment, as previously described, depends on symptoms and blood levels of methemoglobin.

In our case report the only sign of methemoglobinemia was the detection of suboptimal oxygen saturation on pulse oximeter, without evident signs of respiratory distress, so that no specific therapy was performed. We assisted to a progressive resolution of methemoglobinemia after red blood cells transfusions administration.

In our knowledge, even if several authors have reported cases of hemolytic crises due to favism related to a moderate increase in methemoglobin levels, the association with symptomatic methemoglobinemia is rare (Table 3). In detail, Ata et al. described a total of 8 cases of which 6 belonging to previous Literature, in which this coexistence has occurred; all patients were male with an average age of 18 years and only 4 patients belonged to the pediatric population with an average age of approximately 3 years. The median MetHb level detected was 7.8% [5].

**Table 3:** Reported cases of G6PDH deficiency and methemoglobinemia

**Author and Reference**	**Gender**	**Age**
Browning LA and Kruse JA (2005)^c^ [27]	Male	50 years
**Bhat P,et al (2008)**^c^ [28]	Male	12 years
**Schuurman M, et al. (2009)**^**a**^ [6]	Male	1 years
**Bombery MM, et al. (2009)**^c ^[29]	Male	15 years
**Borinstein SC, et al.(2008)**^c ^[30]	Male	14 years
**Fayazi A, et al. (2010)**^c ^[31]	Male	15 years
**Odievre MH, et al. (2011)**^a^ [22]	Male	6 years
Elinoff JM, et al. (2011)^c^ [32]	Male	55 years
Au WY, et al. (2011)^b^ [13]	Male	54 years
**Bauters T, et al. (2011)**^c^ [33]	Male	6 years
Zulqarnain S, et al. (2012)^c^ [34]	Male	56 years
**Ng JS, et al. (2011)**^c^ [35]	Male	16 years
Kang SH, Xie Q (2012)^c^ [36]	Male	56 years
Sonbol MB, et al. (2013)^c^ [37]	Male	52 years
Cheah CY, et al. (2013)^c^ [38]	Male	46 years
Bucklin MH, et al. (2013)^c^ [39]	Male	62 years
**Leunbach TL, et al. (2014)**^**a**^ [23]	2 Male	4 years, 6 years
Zhang B, et al. (2014)^c^ [40]	Male	72 years
**Pansy J, et al. (2014)**^c^ [41]	Male	10 weeks
Dasararaju R, et al.(2014)^c^ [42]	Male	56 years
**Bontant T, et al. (2014)**^c^ [43]	Male	5 years
**Bistolarides J, et al. (2014)**^c^ [44]	Female	15 years
Journal of Hospital Medicine (2015)^a^ [45]	Male	43 years
**Berant R, et al. (2015)**^a^ [46]	Male	9 months
Roberts DA and Freed JA (2015)^c^ [47]	2 Female	70 years, 43 years
Oluwasanjo A, et al. (2015)^c^ [48]	Male	56 years
Sherwood GB, et al. (2016)^c^ [49]	Male	56 years
Reeves DJ, et al. (2016)^c^ [50]	Male	46 years
Montgomery KW and Booth GS (2017)^c^ [51]	Male	50 years
Khan M, et al. (2017)^c^ [52]	Male	73 years
**Cooling L (2017)**^c^ [53]	Male	15 years
**Akande M, et al. (2017)**^c^ [54]	Male	14 years
Rehman A, et al. (2018)^a^ [16]	Male	30 years
Younis M, et al. (2018)^c^ [55]	Male	78 years
Ferguson D and Kovach AE (2018)^c^ [56]	Male	50 years
Makkar P, et al. (2018)^c^ [57]	Male	61 years
Raru Y, et al. (2019)^c^ [58]	Male	56 years
**Leibman M, et al. (2019)**^c^ [59]	Male	1.5 years
Feng C, et al. (2019)^c^ [60]	Female	64 years
Bachmann KF, et al. (2019)^c^ [61]	Male	73 years
Ata F, et al. (2020)^a^ [5]	Male	43 years
Lim S, et al (2020)^b^, ^c^ [10]	Male	39 years
Palmer K, et al. (2020)^b^ [8]	Male	62 years
Kuipers M, et al. (2020)^b^, ^c^ [11]	Male	56 years
Cabantous P, et al. (2020)^c^ [62]	Male	50 years
**Friedman N, et al. (2020)**^c^ [63]	Female	15 years
Ata F, et al. (2021)^a^ [7]	Male	56 years
Al-Dubai H, et al. (2021)^a^ [15]	Male	47 years
**Pomoni A, et al. (2021)**^a^ [21]	Male	3 years
**Pirrone I, et al (2021)**^c^ [19]	Male	14 years
Laslett N, et al. (2021)^b^, ^c^ [12]	Male	60 years
Lopes DV, et al. (2021)^b^ [9]	Male	35 years
Madanat L, et al. (2021)^c^ [64]	Female	91 years
Becker E, et al. (2021)^c^ [65]	Female	85 years
**Lertthammakiatt S, et al (2022)**^a^ [45]	Male	4.5 years
**Kashari OF, et al. (2022)**^b^ [18]	Male	11 years
Ingham S, et al. (2022)^c^ [66]	Male	69 years
Ganapathi M, et al. (2022)c [67]	1 Female, 1 Male	84 years, 20 years
Hammami MB, et al (2023)^c^ [68]	Male	64 years
Malakah, M. A, et al (2023)^b^ [14]	Male	52 years
**Maddumabandara K, et al (2024)**^**c**^ [17]	1 Male, 1 Female	8 years, 40 years

^a^secondary to fava bean ingestion

^b^secondary to infection (HEV, Covid-19)

^c^secondary to drugs administration (Rasburicase, Hydroxychloroquine, Acalypha indica)

**Bold **text pediatric population cases

Al-Dubai et al. [15] described the case of a 47-year-old male patient that, after the ingestion of fava beans, developed a hemolytic crisis together with asymptomatic methemoglobinemia treated with a conservative approach. Similarly, Pomoni et al. and Lertthammakiat et al. presented two pediatric cases (2 males of 3- and 4.5-year-old) who manifested this association of symptomps after consuming fava beans [21].

Some authors highlighted the correlation between hemolytic crises in patients suffering from G6PDH deficiency and methemoglobinemia secondary to drugs exposure. In detail M. Bakri Hammami et al. [68] reported several cases related to the administration of rasburicase, normally used in the management of tumor lysis syndrome (TLS). On this matter Pirrone et al. also described the case of a 14-year-old patient, diagnosed with T-cell acute lymphoblastic leukemia, who developed methemoglobinemia and hemolytic anemia with low oxygen saturation after starting steroids, hyperhydratation, and rasburicase administration [19]. Other case reports showed an association between acute oxidative hemolysis and methemoglobinaemia after the intake of Acalypha indica, normally present in some homeopathic preparations [17]. Regarding infectious triggers, this phenomenon has been reported in a male patient with hepatitis E virus (HEV) infection [13] and, more recently, in case of COVID-19 infection, both with or without the administration of hydroxychloroquine [8–12, 14, 18].

The coexistence of hemolytic crisis in presence of G6PDH deficiency and methemoglobinemia is a phenomenon already described in literature, especially after the ingestion of fava beans. Our case report may represent one of the first example in the pediatric population, potentially related on the one hand to Norovirus infection and on the other hand attributable to ibuprofen administration. In addition, the detection of methemoglobinemia, even if it is not a typical sign of the disease, should always be adequately investigated, because it may potentially hide a condition of underlying G6PDH deficiency. This eventuality should always be excluded, especially before the administration of methylene blue which may cause a worsening of patient’s clinical conditions.

Furthermore, the case we described underscores the fact that neither age at onset nor sex can reliably exclude an underlying G6PD deficiency. Notably, our 7-year-old female patient had an unremarkable personal and family history, which contributed to a delayed and non-immediate diagnosis.

## Data Availability

Not applicable.

## Abbreviations

G6PDH: Glucose-6-phosphate dehydrogenase
RCC: Red cell concentrates
WHO: World Health Organization
PPP: Pentose phosphate pathway
NADPH: Nicotinamide adenine dinucleotide phosphate
GSH: Glutathione
O_2_-Sat: Oxygen Saturation
MetHb: Methemoglobin
uPr: Urinary proteins
uCr: Urinary creatinine
FENa: Fractional excretion of Sodium
TLS: Tumor lysis syndrome
